# Recognition Stage for a Speed Supervisor Based on Road Sign Detection

**DOI:** 10.3390/s120912153

**Published:** 2012-09-05

**Authors:** Juan-Pablo Carrasco, Arturo de la Escalera, José María Armingol

**Affiliations:** Intelligent Systems Lab, Universidad Carlos III de Madrid, Avda de la Universidad 30, 28911 Leganes, Spain; E-Mails: juanpa@ing.uc3m.es (J.-P.C.); armingol@ing.uc3m.es (J.M.A.)

**Keywords:** ADAS, detection, recognition, road signs, pattern matching, neural network

## Abstract

Traffic accidents are still one of the main health problems in the World. A number of measures have been applied in order to reduce the number of injuries and fatalities in roads, *i.e.*, implementation of Advanced Driver Assistance Systems (ADAS) based on image processing. In this paper, a real time speed supervisor based on road sign recognition that can work both in urban and non-urban environments is presented. The system is able to recognize 135 road signs, belonging to the danger, yield, prohibition obligation and indication types, and sends warning messages to the driver upon the combination of two pieces of information: the current speed of the car and the road sign symbol. The core of this paper is the comparison between the two main methods which have been traditionally used for detection and recognition of road signs: template matching (TM) and neural networks (NN). The advantages and disadvantages of the two approaches will be shown and commented. Additionally we will show how the use of well-known algorithms to avoid illumination issues reduces the amount of images needed to train a neural network.

## 1. Introduction

Road traffic accidents are an important socioeconomic problem. As the number of vehicles on the road increases so too does the number of accidents. As a consequence of road accidents, every year approximately 1.2 million people are killed and 50 million are disabled or injured [1]. Not only road are traffic accidents the eleventh cause of death in the World, but it is the only cause of death among the first twelve which is not related to illnesses or diseases, and the picture in the future is bleak, as in comparison to 2003, the number of deaths and casualties worldwide from traffic accidents will increase in 65% [2,3].

Usually car accidents involve speed, which is directly related to an increase in the severity of the accident. Due to this fact, different initiatives have been taken in order to reduce velocity where necessary, these include physical obstacles which force the driver to reduce the speed of the vehicle (such as speed humps or roundabouts) and on-board vehicle technologies that may send useful information to the driver or take control of the vehicle. In this article we present an ADAS whose goal is the speed supervision based on the recognition of road signs and the information of speed given by a GPS-inertial sensor. The system warns the driver in the case that the speed of the car is over a certain limit which is given by the symbol of the road sign.

**Figure 1.**
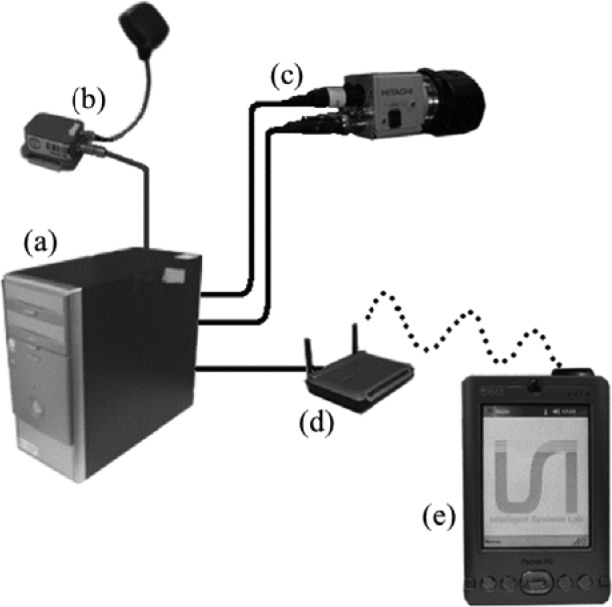
System proposed (**a**) is a PC where the information of the inertial sensor (**b**) and the camera (**c**) are integrated. When necessary, the system sends a message to the driver via wireless connection (**d**) to a PDA (**e**) which will always show an image of the recognized road signs and will broadcast an acoustic message to the driver in case a condition (speed-road sign) is met.

A scheme of the system may be seen in Figure 1. It is composed of a GPS-IMU MTi-G X-Sens device that provides accurate data at 120 Hz of vehicle motion and location. This information is used to obtain the real speed of the vehicle, which is simultaneously compared to the information given by the symbol of the recognized road sign, if there is any in that moment. The color camera used is a Hitachi KPD-20A which is in continuing acquisition of images. These images are processed in the PC to extract the possible road signs and the information contained in their plates. Finally, in the case that the speed of the vehicle is over a certain limit, the driver receives from the PDA a warning acoustic message indicating the maneuver to do and showing the recognized road sign on the screen.

The full recognition of road signs is divided into three stages: detection, recognition and tracking. In the first one, the goal is to detect the objects in the image that have high probability of being road signs. The second one is two-fold: rejection of false positives from the previous stage and extraction of the information of the symbol within the road sign. In the tracking stage a prediction of the location of the road sign is performed in order to assure the results of the recognition. This article will be focus on the stage of recognition, as an extension of the research on detection described in [4]. For this aim, the most spread methods on this field have been used: template matching (TM) and neural networks (NN).

The article is divided into five sections: first we will review previous works regarding recognition of road signs. After this, we will reach the core of the article, which is divided into three parts: the first one explains a required preprocessing stage for both recognition algorithms (TM and NN). This is applied on the input images used to correlate to the templates in the case of TM and to the training samples for the NN approach. The second part explains the use of template matching and neural networks in our system. Results and comparisons of the two approaches are presented in the third main part. Finally the conclusions and future work are described.

## 2. State of the Art

The most widespread methods for recognition of road signs are based on TM and NN, though other classifiers are being introduced lately. Template matching in some cases, as in [5], uses encoded data from images to be correlated with a database of artificial samples, in this case, road signs. But usually the matching is done between gray scale images (template + region of interest) [6–9] using different methods to evaluate the similarity. This measure is usually done using cross correlation techniques [10–12] where a hierarchical approach may also be implemented due to the expense time that usual correlation requires [4]. A similar approach, but in the frequency domain is presented in [13] where a fringe-adjusted joint transform correlation (FJTC) technique is used.

There are many different approaches using neural networks but the more widespread ones are the radial basis functions (RBF) with different activation functions [14–17], and the multilayer perceptron [18–24]. It is difficult to compare the results of these methods since all of them have about 90% positive classification rates. The main differences are related to the source of the training sets: artificial samples [20], real samples [24,25], and a mixture of them, *i.e.*, applying artificial noise or rotations to real images as in [26]. But in the end, the performance is more less the same. The only advantage of artificial sets is avoiding the difficulty of collecting real images for training for all possible road signs.

As a result of this complexity to compare previous works, the results of the following papers, where authors have presented comparisons among different approaches, different NN or different methods within a given approach are very interesting. In [26] there is a comparison among NN, k-NN and Bayesian classifier to recognize road signs using RGB images and real training samples with artificial noise. In [19] we can find a discussion on different NN: MLP, RBF, LVQ and Hopfield NN attending their robustness, precision, and time and memory consuming for circular road signs. A comparative analysis among different classifiers is in [27] where the performance of Laplace, Gauss, mixture of both Laplace and Gauss, ldc, qdc, k-NN is shown. They are able to recognize a large set of road sign's shapes: circles, triangles, diamonds, squares and octagons. A comparison between polynomial classifier and RBF is in [28], where the objective is to maintain the false positive rate as low as possible. They used binarized real images for the training set for circular road sign classification; in [15], random forest with a support vector machine under different conditions along with a comparison of the bagging support vector machines with the AdaBoost naive Bayes approach, has been made. The goal was to recognize 15 different road signs, and for this purpose, they used a set of gray scale images (2,500) for training and testing with sizes of 30 × 30 including non-road-sign images to enhance the rejection capability of the system. Finally a ring partitioned method has been proposed in [29] whose goal is the histogram matching of images. Six different approaches for this tool are presented to achieve the recognition of prohibition and obligation road signs whose size is over 100 pixels.

Finally, also classifiers as support vector machines (SVM) are currently a spread solution for recognition of road signs [27,30–36]. Other approaches use SIFT descriptors [37], PCA [38], naive Bayes [39] or Forest- Error-Correcting Output Code [40]. A presentation of the state of the art for road sign detection can be found in [41].

## 3. Model Preprocessing

Real images always have some level of noise, which affects the results of any classification method. In this section we will apply a number of algorithms in order to obtain better input images for the recognition methods used, TM and NN. These preprocessing algorithms are: (a) based on histogram stretching to mitigate the effects of illumination on the image and (b) based on threshold algorithms to avoid the effects of both illumination and image noise for both the road sing and the background.

**Figure 2.**
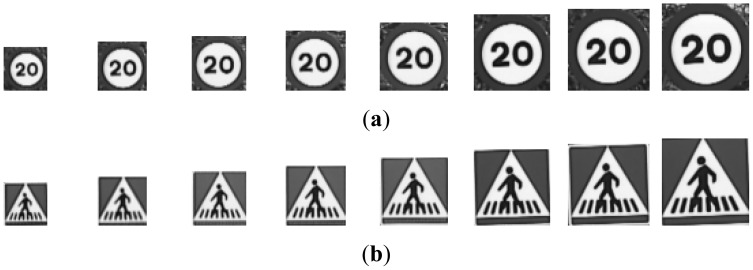
Examples of candidates: (**a**) Red road signs. (**b**) Blue road signs.

There will be five different groups of samples, one for each preprocess applied to the road signs. To make the process understandable for the reader a set of images have been selected and the algorithms will be applied on them. The road sign images to be recognized that have been used are the output of a previous detection stage which is fully explained in [4]. From now on, these images will be called “candidates” and they may be real images of road signs or false detections. The initial set of samples for red and blue road signs may be seen in Figure 2.

**Figure 3.**
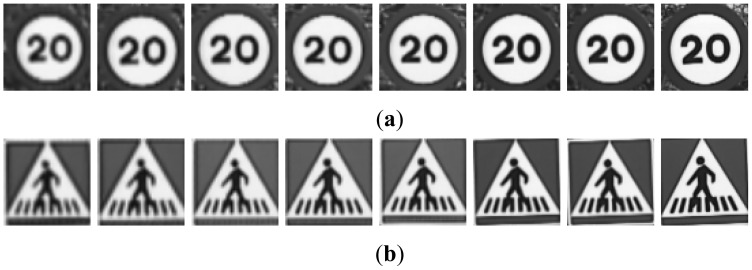
Examples of candidates in Figure 2 after resizing: (**a**) Red road signs. (**b**) Blue road signs.

**Figure 4.**
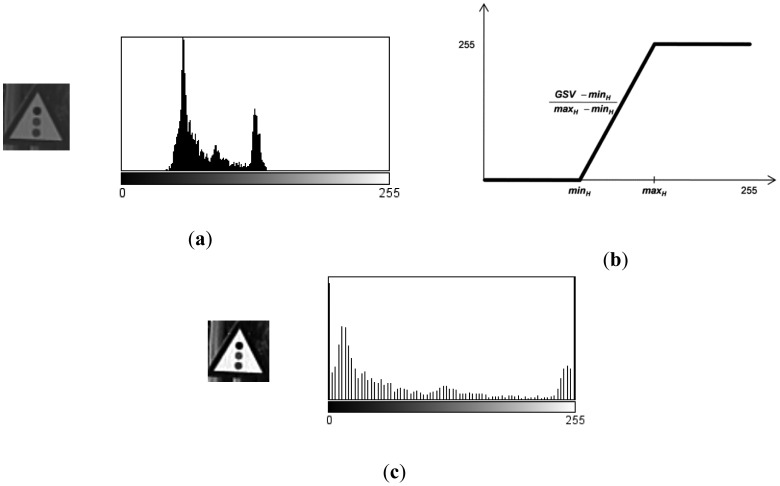
Example of the histogram stretching application (**a**) Gray scale candidate and its histogram. (**b**) Function applied to (**a**) by means of Equation (1). (**c**) Gray scale image and its histogram resulting from histogram stretching.

**Figure 5.**
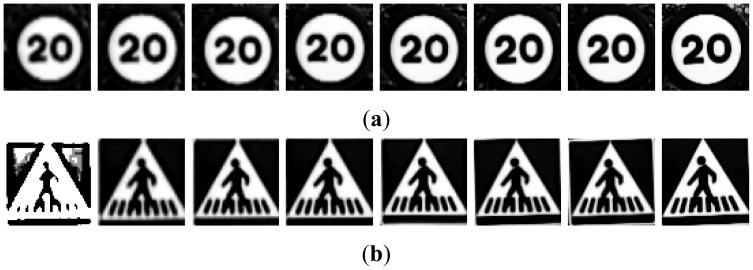
Examples of candidates in Figure 3 when histogram stretching is applied (**a**) Red road signs. (**b**) Blue road signs.

**Figure 6.**
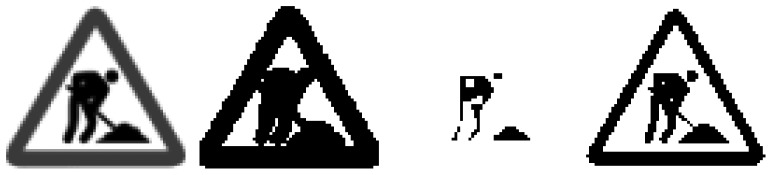
Process to find the optimal thresholding for the P-Tile Method. From left to right: gray scale image, thresholding at GSV = 254, thresholding at GSV = 1, thresholding at GSV = 90.

**Figure 7.**
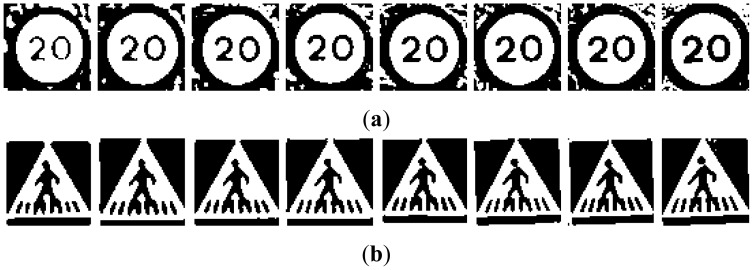
Examples of candidates in Figure 5 processed through a 15% thresholding P-Tile for (**a**) Red road signs. (**b**) Blue road signs.

**Figure 8.**
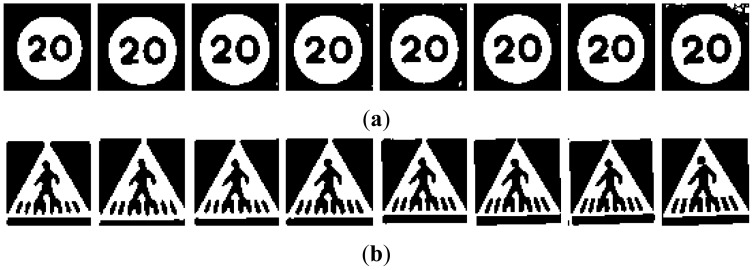
Examples of candidates in Figure 5 processed using Otsu thresholding for (**a**) Red road signs. (**b**) Blue road signs.

**Figure 9.**
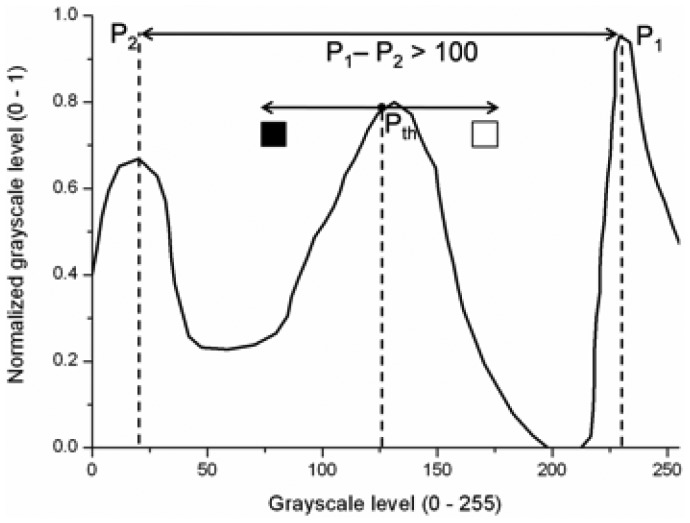
Scheme of the bimodal binarization.

**Figure 10.**
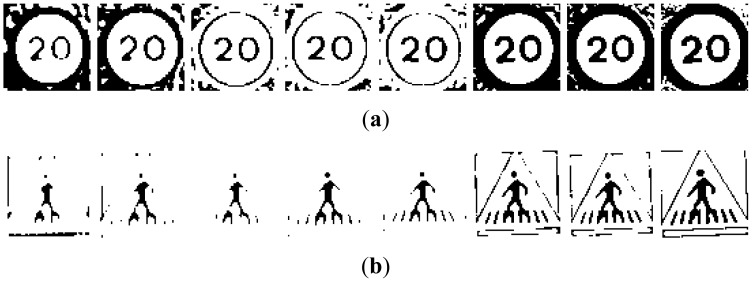
Examples of candidates in Figure 5 processed using bimodal thresholding for (**a**) Red road signs. (**b**) Blue road signs.

The different groups of input images, G1 to G5, are obtained from the candidates in Figure 2:

**G1:** The result of cropping the gray scale image provides the first set of candidates, *i.e.*, the raw gray scale images extracted from the detection stage as may be seen in Figure 3.
**G2:** To obtain the second set of candidates, histogram stretching is performed for each single candidate. The applied function is depicted in Figure 4 and the result over the resized image is in Figure 5. The process is as follows: the histogram of the candidate is computed, min_H_ and max_H_ are each chosen to be equal to 1% of the total amount of pixels in the image. Then we simply assign 0 to those pixels whose gray scale value (GSV) is below min_H_, 255 to those over max_H_ and GSV to those between min_H_ and max_H_. The whole function is described by Equation (1):
  (1) $$\text{GSV}\prime =\{\begin{array}{ccc} 0 & if & \text{GSV}\leq min_{H}\\ 255\cdot \frac{\text{GSV}-min_{H}}{\text{max}-min_{H}} & if & \min_{H}<\text{GSV}<max_{H}\\ 255 & if & \text{GSV}\geq max_{H} \end{array}$$
**G3:** This group is the result of a P-Tile thresholding. For each class, all artificial images have been experimentally evaluated to set a specific binarization threshold, which fits each road sign class. This threshold has been chosen by direct observation of the differences obtained from different threshold levels. An example of this process is presented in Figure 6. It is clearly seen that the threshold level has a noticeable influence in the final binarization.
  The values obtained from the artificial images will be used in the preprocessing of the real candidates and each threshold level will be applied to each corresponding candidate. An example of this may be seen in Figure 7.
**G4:** the fourth set of candidates, depicted in Figure 8, is done using an Otsu thresholding.
**G5:** A bimodal binarization is used to build the fifth set of images. The distance between the reference peaks has been set to 100 gray scale units. This value has been experimentally selected after inspection of the candidates by means of size variation due to distance to the camera, and weather conditions. The middle point between these two reference peaks will give the final thresholding point. Figure 10 shows an example of the process followed using the scheme depicted in Figure 9: the highest peak is taken as the first reference peak, then the maximum peak which is separated by more than 100 gray scale levels is taken as the second reference peak. The medium value of them is the threshold level, which will divide the space into black and white.
  Independently of the method used, TM or NN, the final processing of candidates has been developed by applying two different masks, whose goal is to erase everything but the information inside the plate. Its use is obvious, for TM the influence of the rim of the road sign is neglectable, as the template on which to do the correlation includes the rim. In the case of NN, the method is very sensitive to any addition of spurious information, this is why background and rim erasure is needed.
  In both cases, TM and NN, the application of such a mask to erase the background in one case, and the background and the rim in the other, is straightforward. As result for TM, the pixels belonging to the background after masking will not participate into the calculation of the similarity. In the case of NN, the pixels belonging to the background are set to white to eliminate the influence of the noisy background in the NN learning process.

## 4. Comparison between Template Matching and Neural Networks

### 4.1. Template Matching

**Figure 11.**
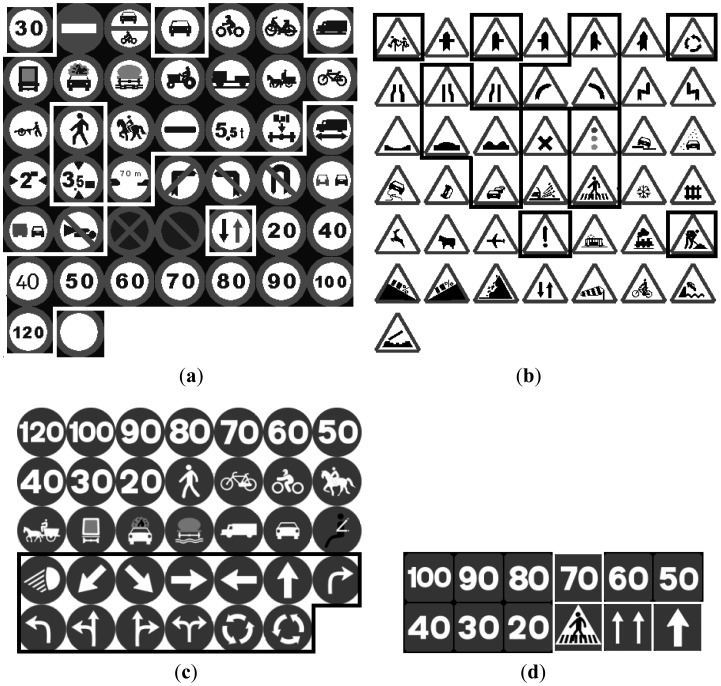
Examples of templates used for recognition by correlation with the candidates. The road signs which are framed correspond to the sets for NNs. (**a**) Prohibition road signs. (**b**) Danger road signs. (**c**) Obligation road signs. (**d**) Indication road signs.

The input candidates from the detection stage were normalized to 64 × 64 and preprocessed to be compared to a template described in Figure 11 where the different road signs are contained. Then each candidate will be moved all over the template and the value of the normalized cross correlation will be used to evaluate the similarity between candidate and template. The experiment has been done separately for red and blue road signs.

### 4.2. Neural Networks

The initial research of this thesis on NN's was developed in the Vislab [42] in Parma, Italy, where the NN used was a LWN++ [20]. This is an open source implementation which uses a general feed forward NN with back propagation training. The neurons of each layer compute synchronously the weighted mean of the outputs of the previous layer and then apply the activation function to the result, which is given by Equation (2). Only the logistic part of the activation function has been used providing results between 0 and 1:

(2) $$f(x)=\frac{1}{1+e^{-x}}$$

The input samples are 64 × 64 pixel images which, by means of shape, are inserted in the corresponding NN. To breakdown the recognition problem it is possible to use some specialized NN with a few outputs instead of using a big one with many outputs. Five different NNs have been developed, which correspond to Danger, Yield, Prohibition, Obligation and Indication road sings. A hidden layer has also been added where the number of neurons is modified to obtain the best performance of the net. Regarding the training of the NN, it is necessary to find real samples of road signs, and moreover, it is necessary to find a large enough set of them also to validate the results. This search is not a straightforward task and as a result, the total number of road signs that can be recognized using our NN is 54, much less than the 135 using the TM approach, see Figure 11.

For this reason the goal is to reduce the amount of samples used for training and validating. Let's see Figure 8. The data that remains after preprocessing (resizing + thresholding) is more than sufficient to train the NN and it is almost the same for the distances in which our system can detect a road sign. Then, instead of using all those candidates to train the net, it is enough using just one. The advantages are fourfold: the sets for training and validation are more easily built, the time expended in training and validating decreases, the simplicity for the user to manually select the candidates for the training set increases, and there is a decrease of the risk of redundancy.

**Figure 12.**
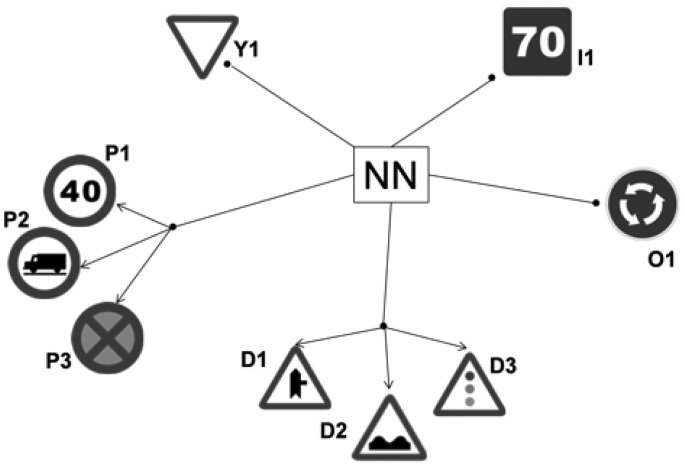
Examples of the output of the cascade network for road sign recognition. Each figure shows the group it belongs to.

Two approaches were made for NN: First, a Single-Stage Neural Network (SSNN), in which only a NN is implemented for each class (Danger, Yield, Prohibition, Obligation and Indication road sings). Secondly, the cascade approach (CNN) simplifies the problem of recognition by arranging smaller groups of road signs within a certain class. The road signs that were usually wrong classified using the SSNN are inputs for a second NN. This results in more specialized subnetworks and so it is expected that the number of incorrect recognitions will decrease. The groups for this approach are in Figure 12 and listed here:

- **Yield. Y1:** In this case, since there is only one output it is not necessary to use the cascade approach, the network is exactly the same as in the SSNN case.
- **Prohibition. P1:** Speed limit road signs. **P2:** No trucks. **P3:** Parking, Stop and Driving.
- **Danger. D1:** Merging. **D3:** Uneven road, Hump. **D4:** Other dangers, traffic lights.
- **Obligation. O1:** Roundabout.
- **Indication. I1:** In this case no cascade approach has been developed as there were no confusions among members.

## 5. Results and Discussion

### 5.1. Template Matching: Red Road Signs

In Table 1, the recognition results using TM for red candidates may be seen. The true positive rate (TP_rate_) takes into account the rate of recognition over the total amount of true detections. True detections stands for the samples from the detection stage that were correctly classified as road signs. The false positive rate (FP_rate_) is the rate of false recognitions and incorrect recognitions over the total amount of detections. It may be seen that the best performance corresponds to the raw gray scale candidates, this is followed by the histogram stretching and finally the Otsu thresholding which is also observed to obtain satisfactory results, 6% and 9% less than previous case. In general, the option for binarization of candidates is not effective for template matching. The normalized cross correlation severely punishes a strong difference of the pixel values and this is why gray scale candidates are obtaining better results.

In general the problems associated with template matching for recognition are related to the FP_rate_ that is very high for all preprocesses that have been used. In order to reduce the amount of false positives, it has been tried to set a threshold for the value of the cross correlation, as a criteria to distinguish between road sign and non-road sign. Unfortunately it is not possible to avoid all these false positives: in Table 2 it may be observed the results on TP_rate_ and FP_rate_ for the first interval. Rejecting candidates within these limits would mean missing a high percentage of true recognitions. For example, in the case of raw gray scale, we could avoid every single false positive if we go up with the threshold to 30%, but then we would miss 58% of the true recognitions.

### 5.2. Template Matching: Blue Road Signs

The results for recognition using template matching of blue road signs are presented in Table 3. In the case of blue road signs, the results would not be valid for a recognition system since the true positive rate is very low. These road signs are easily missed, especially in two cases: first, obligation road signs, they often have a more inhomogeneous road sign background than those with a white background, resulting in a low value of the normalized cross correlation. Secondly, the small stripes in the crosswalk road signs are very unstable because of slight movements of the camera or rotations of the road sign, which often cause the system to fail in the recognition.

### 5.3. Neural Networks

For this approach, only two of the four preprocessing treatments have been used: Otsu and P-Tile. As one of the goals of the preprocessing is noise elimination, it made sense not to include gray scale images in the training of the NN. The bimodal algorithm was discarded because of the high dependence of the results on the illumination of the image. Therefore the focus will be on the two remaining binarization methods.

The most significant results for the NN approach are in Tables 4 and 5. In Table 4 we find the results for the SSNN, which are poor, the true recognition rate is very low and the false classification very high. In order to divide the problem, a NN for each road sign's shape had been implemented, but due to the amount of road signs to be classified into each group, and most of all, the high similarity among some of them, the approach failed.

In Table 5 we can see that the performance increases its values especially in the case of the samples preprocessed with the P-Tile method. The results for blue road signs are perfect, though unfortunately the precision is too low due to the high amount of samples that not being road signs were classified as them. Again, as in the case of the TM matching approach, it has been impossible to set a rejection threshold to reduce this misclassification while maintaining a high positive rate of classification. Taking into account the results obtained, the best performance in general is for the cascade NN approach using the P-Tile preprocess. Very similar results are obtained for the template matching approach in the case of red road sign recognition but unfortunately results with this method regarding blue road signs is very poor. The FP_rate_ for both TM and NN is the same 25%. Nevertheless, the trials using our experimental platform IVVI [43] under real conditions gave very good qualitative results, the number of false alarms was very low, even when the speed threshold was turned off in order to test the system in the worst scenario possible. It has to be emphasized that the system performs the three stages (detection, recognition and tracking) in real time, and sends the warning messages in advance so the user can decide the most suitable maneuver. The system was tested under real conditions of illumination. The test sequences were randomly chosen and they correspond to both urban and non-urban environments. In Spain, in urban environments, location and maintenance of road signs do not follow any general rule, so we can find damaged and aged road signs almost anywhere, which increases the difficulties of the task.

## 6. Conclusions and Future Work

This article has shown the comparison of the most spread methods used for classification of road signs: template matching and neural networks. Different preprocessing algorithms for thresholding and binarization have been applied on the gray scale samples to improve results in both approaches. To obtain better neural networks results in positive classification, a cascade configuration has been implemented, rising the results to high quality standards, for the P-Tile algorithm using cascade NN we reached 87% true classification for red road signs and 100% blue road signs while the classification of false positives occurred 26% of the times, which is a very good result taking into account the scenario in which the system is used. Both approaches TM and NN are failing in the rejection of false positives for two cases: misclassification of the true positives from the detection stage and classification of false positive detections.

Both approaches have implementation advantages and disadvantages. The main advantage of the TM approach is how easy it is to add a new road sign to the recognition set, while the addition of one more road sign to the NN imply a new training of the net and manual selection of the training samples, even when in this latter the process is much more eased thank to the preprocessing used, we still have to create a validation set which is usually hard since some of the road signs are difficult to find in a real environment.

The future work should be mainly based in two improvements: quality of the input images and capacity of rejection of the NN. In the first case, the changes in illumination cause that the preprocessing algorithms fail, or at least they do not perform as well as they could. Developing an algorithm for thresholding, whose dependence on the illumination conditions were low, would be an important breakthrough.

Secondly, the addition of an output in the NN to reject the false candidates from the detection stage would help getting better results. This is of course a very difficult task due to the large amount of objects in real environments that look like road signs.

Finally, an open database of sequences recorded by the different national and international research groups would be of great advantage for the development of the road sign recognition systems. This global thinking would help to solve this problem not only locally but also worldwide.

## Figures and Tables

**Table 1.** Results on recognition of red road signs regarding the processing applied to the candidate.

**Template Matching. Red Road Signs**			

Group	Processing	TP_rate_	FP_rate_
G1	Raw Gray scale	0.83	0.25
G2	Histogram Stretching	0.77	0.22
G3	P-Tile	0.75	0.34
G4	Otsu	0.76	0.34
G5	Bimodal	0.27	0.10

**Table 2.** True and false positives in the first interval (20–30)% of correlation for the best preprocessing treatment.

**Processing**	**FP_rate_**	**TP_rate_**
Raw Gray scale	1.00	0.58
Histogram stretching	0.27	0.50
P-Tile	0.84	0.65
Otsu	0.71	0.58

**Table 3.** Results on recognition of red road signs regarding the processing applied to the candidate.

**Template Matching. Blue Road Signs**			

Group	Processing	TP_rate_	FP_rate_
G1	Raw Gray scale	0.45	0.44
G2	Histogram Stretching	0.29	0.16
G3	P-Tile	0.42	0.30
G4	Otsu	0.31	0.22
G5	Bimodal	0.03	0.00

**Table 4.** Results of the SSNN approach for recognition.

**Neural Networks Single Stage Approach**			

Preprocessing	Class	TP_rate_	FP_rate_
Otsu	Red	0.23	0.81
	Blue	0.48	0.80

P-Tile	Red	0.28	0.68
	Blue	0.47	0.59

**Table 5.** Results of the cascade approach for recognition.

**Neural Networks Cascade Approach**			

Preprocessing	Class	TP_rate_	FP_rate_
Otsu	Red	0.52	0.56
	Blue	1.00	0.59

P-Tile	Red	0.87	0.26
	Blue	1.00	0.26
